# *MembraneDyn*: simulating the dynamics of supported membrane stacks on the nanosecond timescale

**DOI:** 10.1107/S2059798322008701

**Published:** 2022-09-27

**Authors:** Dominic W. Hayward, Sebastian Jaksch, Margarita Fomina, Purushottam S. Dubey, Henrich Frielinghaus, Olaf Holderer, Michael Monkenbusch

**Affiliations:** a Jülich Centre for Neutron Science, Forschungzentrum Jülich, Lichtenbergstrasse 1, 85747 Garching, Germany; Oak Ridge National Laboratory, USA

**Keywords:** dynamical simulations, materials modelling, grazing-incidence diffraction, neutron spin-echo spectroscopy, supported membrane stacks, time-dependent intermediate scattering function

## Abstract

The static structure factor and the undulation dynamics of a solid-supported membrane stack have previously been calculated by Romanov and Ul’yanov [Romanov & Ul’yanov (2002). *Phys. Rev. E*, **66**, 061701]. Based on this prior work, the calculation has been extended to cover the membrane dynamics, *i.e.* the intermediate scattering function as a Fourier transform of the van Hove correlation function of the membrane stack. Fortran code which calculates the intermediate scattering function for a membrane stack on a solid support is presented. It allows the static and dynamic scattering functions to be calculated according to the derivation of Romanov and Ul’yanov. The physical properties of supported phospholipid bilayers can be examined in this way and the results can be directly compared with results obtained from grazing-incidence neutron spin-echo spectroscopy experiments.

## Abstract 2

A computational method for obtaining the time-dependent intermediate scattering function of supported membrane stacks is presented.

## 1. Introduction

Multilamellar lipid assemblies play many important roles in living systems. Examples include increasing the volume concentration of protein complexes [as in mitochondrial cristae (Fontanesi, 2015 ▸) or in the thylakoid stacks in chloro­plasts (Mustárdy *et al.*, 2008 ▸)], providing electrical insulation (for example in the myelin sheaths around axons; Bean, 2007 ▸) and regulating the structural and permeability properties of tissue (such as in the stratum corneum of the skin; Iwai *et al.*, 2012 ▸). Multilamellar structures are also used *in vitro*, commonly supported by a rigid substrate, to study various biophysical phenomena such as membrane swelling (Kuklin *et al.*, 2020 ▸), membrane fusion (Pompeo *et al.*, 2005 ▸) and interactions between biomembranes and drug molecules (Jaksch *et al.*, 2015 ▸; Mangiapia *et al.*, 2017 ▸). Aside from their use as tools to study naturally occurring membrane stacks, supported multilamellar assemblies are also finding an increasing number of practical applications, for example in disease diagnosis (Sloan *et al.*, 2013 ▸), cell sensing (Minner *et al.*, 2014 ▸) and drug delivery (Joo *et al.*, 2013 ▸), and have shown potential as catalytic substrates (Heath *et al.*, 2017 ▸) and as tuneable photonic crystals (Lenhert *et al.*, 2010 ▸). Such applications rely on a comprehensive understanding of the structure and dynamics of multilamellar systems and the biophysical processes that underpin them. Further advancement in this field is therefore inextricably linked to the development of experimental and theoretical models that describe them.

The collective dynamics of multilamellar membrane structures are well suited to investigation by scattering methods. In techniques such as dynamic light scattering (DLS), neutron spin-echo spectroscopy (NSE) and X-ray photon correlation spectroscopy (XPCS), the dynamics of the samples are probed as a function of both energy (*i.e.* the timescale of the dynamics) and momentum transfer. This allows the observed fluctuations to be characterized according to the length scales on which they occur. The NSE technique, for example, enables the investigation of relaxation times between 10 ps and 100 ns at *q*-vectors in the region covered by small-angle neutron scattering (SANS), *i.e.* between 0.02 and ∼0.5 Å (Holderer & Ivanova, 2015 ▸); this corresponds approximately to the energy and length scales of membrane collective undulation modes (Kelley *et al.*, 2019 ▸). The dynamics on smaller time and length scales (*i.e.* the motion of individual molecules) can be studied using inelastic neutron scattering (Rheinstädter *et al.*, 2004 ▸). For dynamics with longer relaxation times and larger length scales (for example capillary waves along a phase boundary), XPCS or DLS may be used (Sikharulidze *et al.*, 2002 ▸; Sinha *et al.*, 2014 ▸). By employing several complementary techniques on the same system, a holistic picture of the dispersion relation may be constructed (Rheinstädter *et al.*, 2006 ▸). By combining XPCS and NSE, surface fluctuations in smectic membranes have been studied over a broad range of length scales and timescales to study capillary waves, separating the dynamics in the normal and in-plane directions (Sikharulidze *et al.*, 2003 ▸). Recently, grazing-incidence neutron spin-echo spectroscopy (GINSES) has been shown to provide additional information on the dynamics of supported bilayers on larger length scales of up to 1 µm. GINSES measurements on a membrane stack of phospholipid membranes revealed an in-plane oscillatory mode that had not previously been observed in multilamellar soft matter (Jaksch *et al.*, 2017 ▸). These modes were subsequently also observed with grazing-incidence small-angle neutron scattering (GISANS; Jaksch *et al.*, 2019 ▸).

In order to interpret the results from the scattering techniques outlined above, it is necessary to have a sound theor­etical description of the underlying physical phenomena, as well as a means of linking this theoretical basis to the experimental observations. In the case of supported multilayer systems, the theoretical basis is provided by the work of Romanov and Ul’yanov, hereafter referred to as the Romanov model (Romanov & Ul’yanov, 2002 ▸). Originally conceived to characterize the behaviour of supported, liquid-crystalline smectic films, the Romanov model describes both the fluctuation spectrum, as well as the associated scattering, from a system of discrete layers adjacent to a solid support. The results from this comprehensive work have been used to interpret experimental data concerning the undulation amplitudes (Khondker *et al.*, 2017 ▸) and undulation frequencies (Brotons *et al.*, 2005 ▸) of lipid bilayer systems and also to validate alternative models describing the dynamics of supported multilamellar systems (Constantin *et al.*, 2003 ▸).

In this work, the rigorous theoretical framework of Romanov and Ul’yanov has been extended into the time domain and further developed into the *MembraneDyn* software. The software enables the calculation of both the static and dynamic structure factors and can be used to interpret experimental scattering data from supported soft multilayer systems. We first introduce the mathematical framework behind the calculations, then briefly discuss the implementation and finally assess the effects of various input parameters on the final results and discuss how this information could be used in practice.

## 2. Theory

### 2.1. The Romanov model

A full description of the Romanov model can be found elsewhere (Romanov & Ul’yanov, 2002 ▸); however, it is useful to reiterate the main points here. The formulation is based on a system of *N* discrete parallel layers equally spaced by a distance *d*_layer_, with a free energy given by the surface integral, 

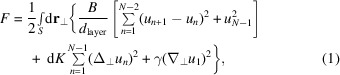

where *u*_*n*_ is the (scalar) *z*-displacement (orthogonal to the substrate) of layer *n* at point **r** **_⊥_** in the *xy* plane, *B* and *K* are the layer compression and elastic constants, respectively, γ is the surface tension, and the integral is a surface integral.

**Figure 1:**
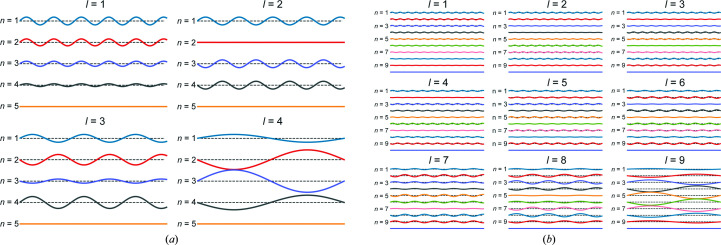
Comparison of the undulation amplitudes and eigenfrequencies ω^*l*^ for different layers *n*, modes *l* and total layers *N* for a given *q*_⊥_. The wavelengths in (*a*) and (*b*) are normalized such that ω^*l* = *N* − 1^ = 1; the undulation amplitudes are likewise normalized to the largest amplitude in *l* = *N* − 1. The colours are not significant other than to distinguish neighbouring layers.

In this geometry, layer *N* corresponds to the fixed substrate (*i.e.* *u*_*N*_ = 0) and layer 1 is the free surface. Under the assumption that the motion of layer *n* arises only due to the elastic force, −*d* ^−1^(δ*F*/δ*u*_*n*_), and the viscous force, η_3_Δ_⊥_(∂*u*_*n*_/∂*t*) (where η_3_ is the layer sliding viscosity), a set of equations can be constructed defining the motion of each layer. If one additionally assumes that the extent of the layers is infinite in the directions parallel to the substrate, and that the motion is governed by plane waves of the form

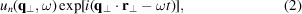

a 2D Fourier transformation yields a set of linear homogeneous equations that can be solved to give the eigenmodes of the system (*i.e.* the eigenfrequencies 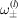 and layer displace­ment amplitudes 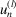 for each mode *l*). In the original work, these equations are solved analytically in the limiting cases of 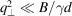 and 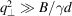 using Chebyshev polynomials. In this work, the roots are found numerically for all values of *q*_⊥_. The eigenmodes for a system of four layers and nine layers (in addition to the immobile substrate) are shown in Fig. 1 ▸.

The spatial correlation functions are obtained via the free-energy expression in equation (1). This can reformulated in Fourier representation as

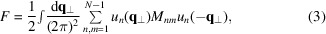

where *M*_*nm*_ are the matrix elements of the tridiagonal matrix 

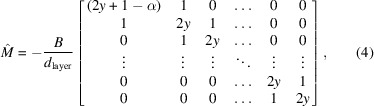

where

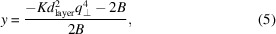




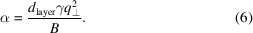




Note that equation (4) arises directly from the underlying set of linear homogenous equations describing the motion of the layers. To a first approximation, each layer interacts only with the layers above and below, giving rise to the tridiagonality when written in matrix form. The spatial layer displacement correlation function is then given by 

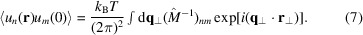




In the original work, the correlation functions are again solved using Chebyshev polynomials. Finally, the expected scattering intensity is determined by calculating the atom positions tagged by their X-ray (electrons) or neutron (nuclei) scattering lengths (*i.e.* the scattering length density convolved with its inverse). This is equivalent to calculating the Patterson function for the membrane stack

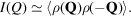




The scattered intensity is therefore given by

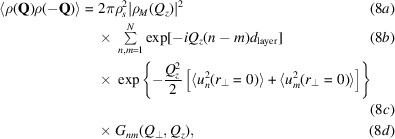

where ρ_*s*_ is the area density of molecules in the layers and ρ_*M*_ is the Fourier transform of the scattering length density (SLD) of the molecules along the *z* axis. Note that although they share the same physical units and indeed are both referred to by the same symbol in the original model, the two quantities *Q* and *q* are distinct and should not be confused. The lower-case *q* is the wavevector associated with the plane waves introduced in equation (2) and forms the variable of integration in equation (3). The upper-case *Q* is the scattering vector, which is an experimental variable. In the formulation above, equation (8*a*) gives the contribution from the scattering length density contrast, equation (8*b*) gives the contribution from the layer–layer distance, equation (8*c*) gives the contribution from the mean-squared displacement and equation (8*d*) gives the layer displacement correlation function, 

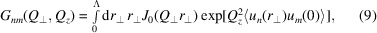

where Λ is the spatial extent of the film surface. Details of how these equations have been implemented and extended into the time domain are provided in the supporting information.

## 3. Results

**Figure 2:**
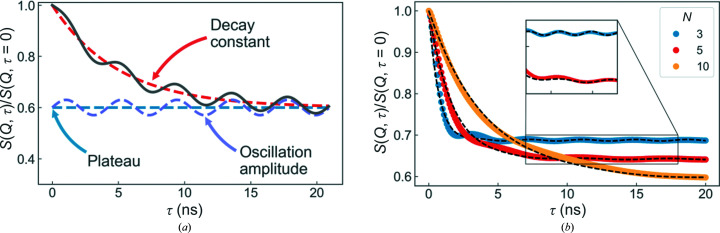
(*a*) Illustration showing the contributions of the terms *A*, *B* and β (dashed lines) to *S*_fit_ in equation (10) (solid line). (*b*) Representative computed intermediate scattering functions, *S*(*Q*, τ), using the parameters given in Table 1 ▸ for *N* = 3, 5 and 10 (circles). The computed data were subsequently fitted with equation (10) (dashed lines).

Due to the large number of experimental variables that are present in the model, it is instructive to examine the effects of each in turn. In this way, it is possible to build up a comprehensive picture of how each parameter affects the dynamics of supported lamellar systems. To aid the interpretation, a selection of the simulated intermediate scattering functions were also fitted using a simple exponential model with an oscillating component of the form 

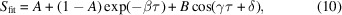

where *A* is the magnitude of the plateau, *B* is the amplitude of the oscillation, β is the decay constant, γ is the relative frequency of the oscillation and δ is the phase of the oscillation. Fig. 2 ▸ shows a graphical representation of these parameters along with examples of the fits to simulated intermediate scattering functions. Although this simple model does not replicate the simulated data perfectly, it is sufficient to identify the sets of parameters of most interest for further experimental study (for example a slow decay with strong oscillations). Unless stated otherwise, the parameters used to generate the results in the remainder of this section are given in Table 1 ▸.

### 3.1. Scattering vectors: *Q*_⊥_ and *Q*_*z*_

**Figure 3:**
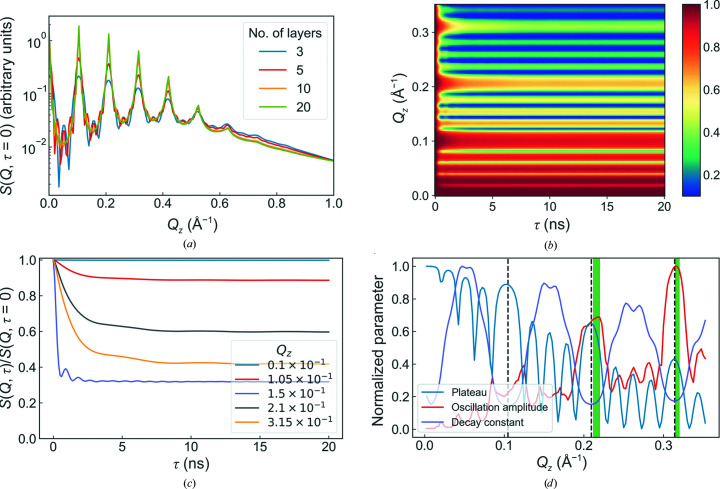
Overview of the simulation results for different out-of-plane scattering vectors. (*a*) The scattering intensity at τ = 0 for different total numbers of layers. The evolution of the intermediate scattering functions with changing *Q*_*z*_ is shown in (*b*) for all data and in (*c*) for selected examples. (*d*) The corresponding evolution of the fitted values. The shaded green bar in (*d*) highlights the region in which the collective dynamic behaviour is observed most clearly. The standard parameter values used can be found in Table 1 ▸.

The scattering vectors link the intermediate scattering function, and hence the dynamics, to the length scales on which they are observed. For the out-of-plane scattering vector *Q*_*z*_, the dynamics are very sensitive to the location of the correlation peaks, as can be seen in Fig. 3 ▸. The height of the plateau gradually decreases with increasing *Q*_*z*_ whilst oscillating with the Kiessig fringes and correlation peaks. The decay constant follows a series of troughs and peaks, where the former coincide with the correlation peaks. This slowing down of the dynamics at the correlation peaks in *S*(*Q*) is known as de Gennes narrowing and has been well documented (De Gennes, 1959 ▸; Holderer *et al.*, 2007 ▸; Sobolev, 2016 ▸; Wu *et al.*, 2018 ▸). Crucially, the amplitude of the oscillations in the intermediate scattering function also exhibits maxima around the correlation peaks. Presumably, this is due to the fact that at these *Q*-values one is explicitly probing the average layer–layer separation distances and therefore layer–layer correlation functions. A corollary of this effect can be be found when examining the behaviour of the inter-layer spacing at constant *Q*_*z*_. The results are shown in the supporting information and highlight the sensitivity of the system to very small changes in sample thickness. For the purposes of model validation, the optimum *Q*_*z*_ value would be on the shoulder of a higher-order Bragg peak; here, the oscillations of the intermediate scattering function (ISF) are still strong and the dynamics are sufficiently fast that long Fourier times are not required.

**Figure 4:**
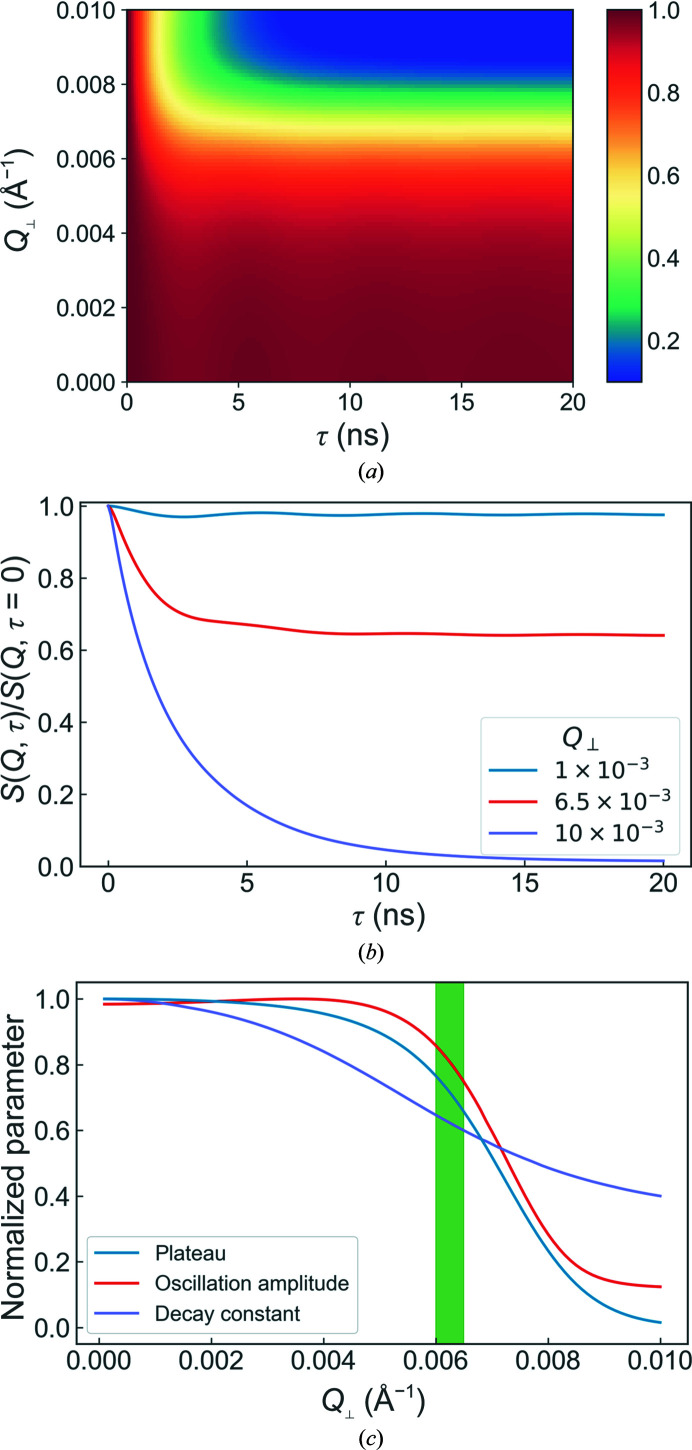
Overview of the simulation results for different in-plane scattering vectors. The overall evolution of the intermediate scattering functions with changing *Q*_⊥_ and some representative examples are shown in (*a*) and (*b*), respectively, whilst (*c*) shows the corresponding evolution of the fitted values. The shaded green bar highlights the region in which the collective dynamic behaviour is observed most clearly. The standard parameter values used can be found in Table 1 ▸.

The behaviour of the ISF with increasing *Q*_⊥_ is shown in Fig. 4 ▸. At small in-plane scattering vectors the plateau remains close to unity with comparatively strong oscillations. Conversely, for large in-plane scattering vectors the ISF decays to a very low plateau with little oscillation. The transition between the high-plateau/strong-oscillation and low-plateau/weak-oscillation regimes occurs at *Q*_⊥_ ≃ 0.005–0.008 Å^−1^. This threshold marks the approximate boundary below which collective behaviour is observed and is thought to correspond to the wavelength of the dominant membrane oscillations, in this case ∼80 nm. Interestingly, this corresponds almost exactly to the wavelengths of 75–100 nm observed experimentally for collective oscillations in a lipid membrane stack via GISANS (Jaksch *et al.*, 2019 ▸).

### 3.2. Number of layers: *N*

**Figure 5:**
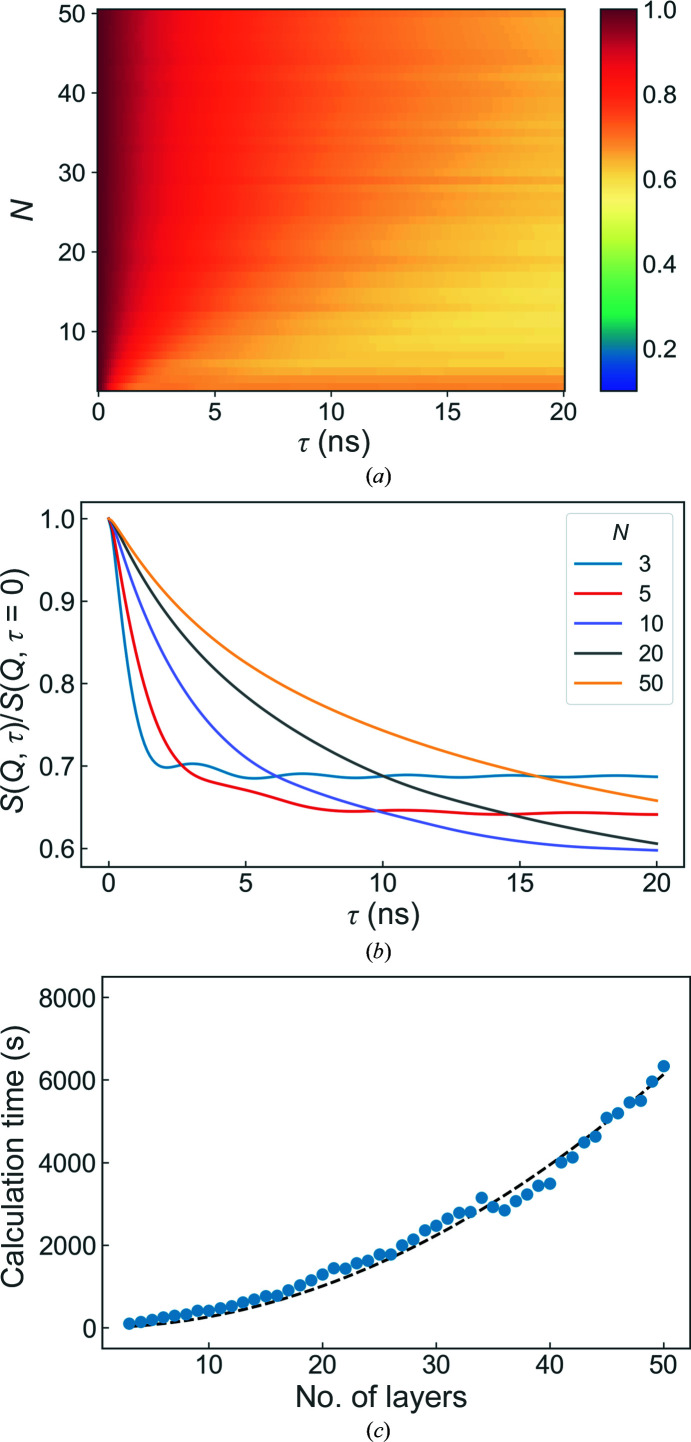
Overview of the simulation results for different values of *N*, the total number of layers. The overall evolution of the intermediate scattering functions with changing *N* and some representative examples are shown in (*a*) and (*b*), respectively. (*c*) shows the time taken for each calculation (points) and the expected *t* ≃ [*N*(*N* + 1)]/2 dependence (dotted line). The standard parameter values used can be found in Table 1 ▸.

The effects of increasing the number of layers in the system is shown in Fig. 5 ▸. The number of lamellae in a stack principally influences the observed dynamics in two ways. Firstly, and rather trivially, the number of layers affects the static structure factor *S*(*Q*, 0), as shown in Fig. 3 ▸(*a*). Increasing the number of layers gives rise to sharper Bragg peaks and more fringes in the structure factor. As shown above, the scattering function *S*(*Q*, τ) is rather sensitive to the scattering vector being probed (*i.e.* the proximity of *Q*_*z*_ to a peak in the static structure factor). However, this sensitivity can be somewhat mitigated by probing at a scattering vector on the shoulder of a Bragg peak.

Secondly, the number of layers affects the dynamics of the system as a whole: the more layers that are present, the greater the number of available energy modes. Importantly, this means that the undulation amplitude of the layers closest to the solid surface varies in a discrete (and not necessarily linear) fashion. The effect of this is that the dynamical behaviour does not vary linearly with the number of layers, as can be seen from the darker and lighter stripes in Fig. 5 ▸(*a*). This can be problematic for experimental systems, where the precise number of layers is not necessarily well known or constant over the illuminated sample. In general, however, it can be seen that increasing the number of layers has the effect of damping the collective dynamics (lower oscillation amplitudes), slowing the overall dynamics (smaller decay constants).

It should also be noted that increasing the number of layers in the system increases the calculation time; this can be seen in Fig. 5 ▸(*c*). On a single core, the calculation time of one ISF for a five-layer system is approximately 180 s. The calculation time scales with *t* ≃ [*N*(*N* + 1)]/2, a dependence which stems directly from the total number of correlation functions *u*_*nm*_ that must be calculated in a system with *N* layers. For larger systems, the calculation times were observed to increase faster than this triangular scaling, most likely due to bottlenecks associated with the storage and manipulation of very large arrays.

### 3.3. Layer compression modulus: *B*

**Figure 6:**
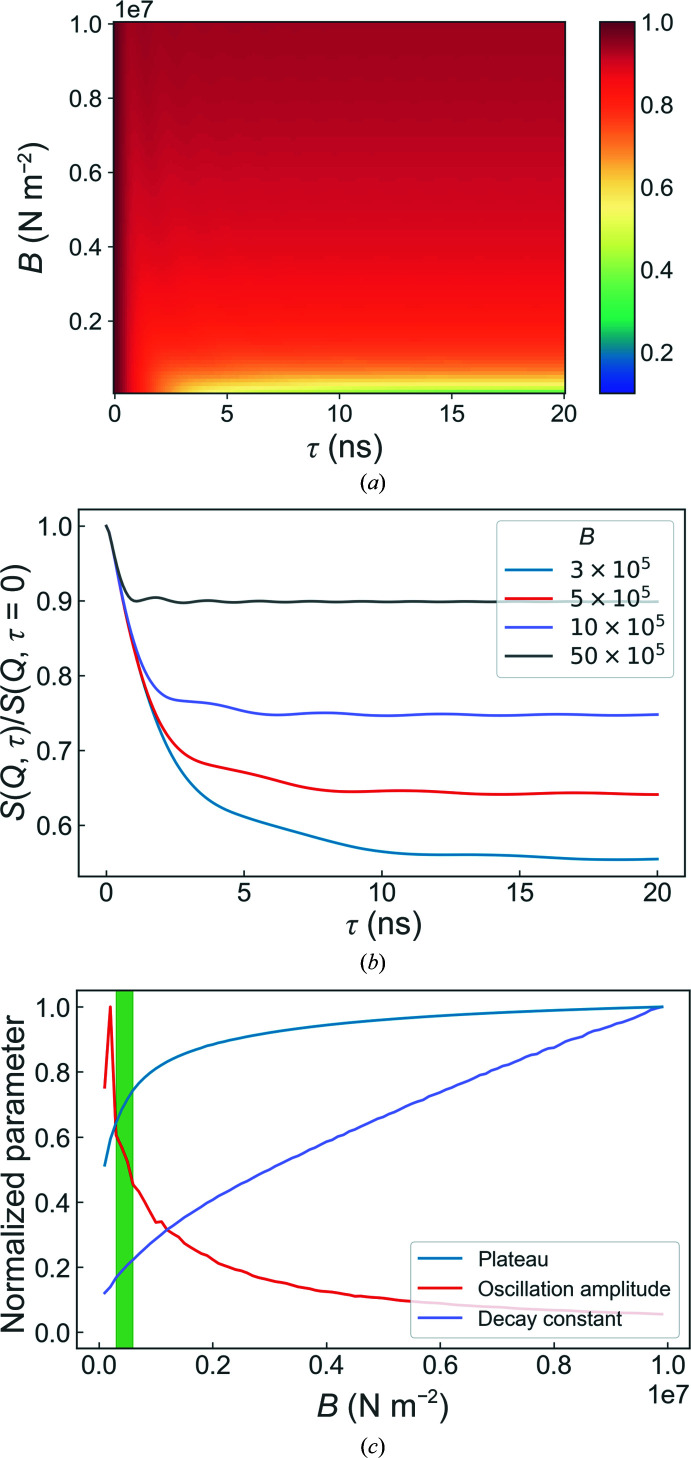
Overview of the simulation results for different layer compression moduli. The overall evolution of the intermediate scattering functions with changing *B* and some representative examples are shown in (*a*) and (*b*), respectively, whilst (*c*) shows the corresponding evolution of the fitted values. The shaded green bar highlights the region in which the collective dynamic behaviour is the most stable. The standard parameter values used can be found in Table 1 ▸.

The layer compression modulus describes the ability of a layer to resist changes in area; the higher the compression modulus, the ‘stiffer’ the layer. In lipid multilayer systems, the compression modulus is linked both to the composition of the layer (Saeedimasine *et al.*, 2019 ▸) and to the hydration of the headgroups (Binder & Gawrisch, 2001 ▸), and therefore it is very useful to determine when characterizing a multilayer sample. The dynamical behaviour as a function of the layer compression modulus is shown in Fig. 6 ▸. It can be seen that the amplitude of the ISF oscillations decreases and the plateau height increases as the compression modulus of the membranes is increased. This weakening of the dynamical features is expected, as stiffer membranes will undergo less deformation at a given thermal energy than softer membranes. In Fig. 6 ▸(*b*) it can also be seen that the frequency of the oscillations increases with increasing compression modulus.

### 3.4. Layer sliding viscosity: η_3_

**Figure 7:**
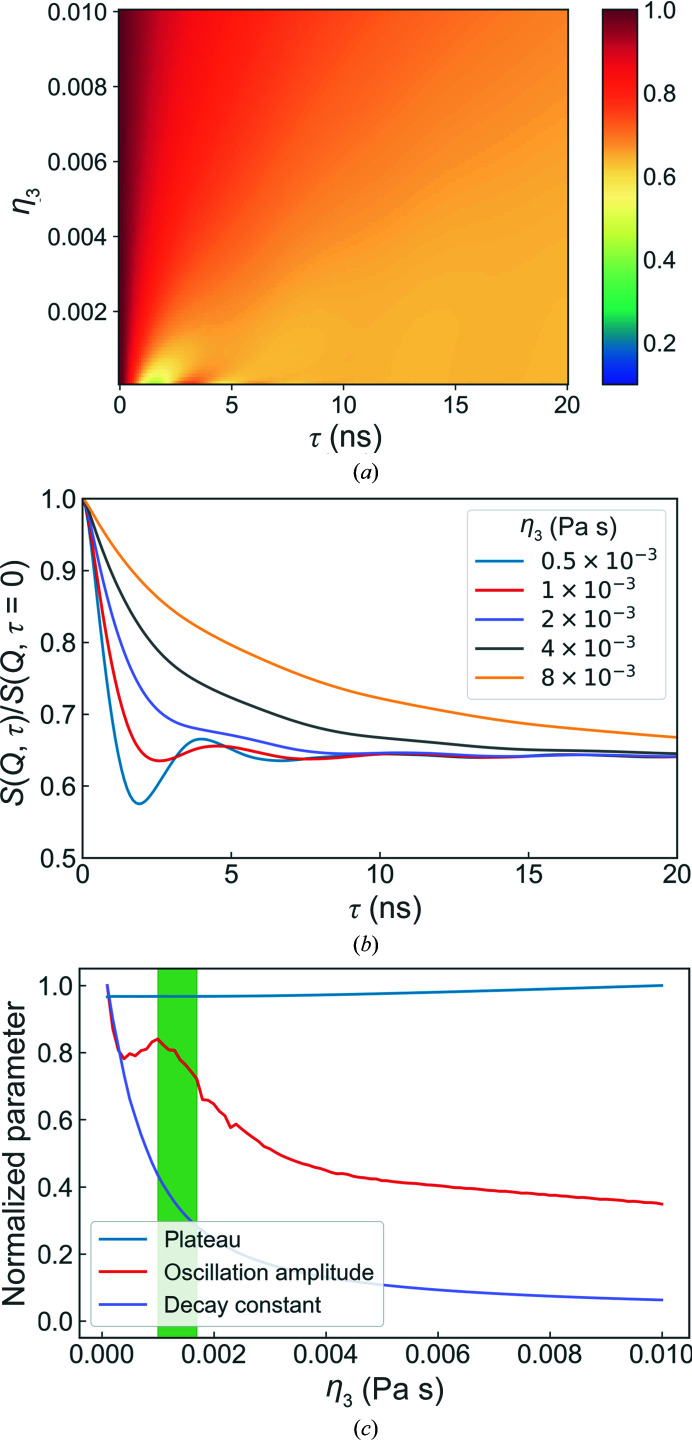
Overview of the simulation results for different layer sliding viscosities. The overall evolution of the intermediate scattering functions with changing η_3_ and some representative examples are shown in (*a*) and (*b*), respectively, whilst (*c*) shows the corresponding evolution of the fitted values. The standard parameter values used can be found in Table 1 ▸.

The layer sliding viscosity, which determines the interlayer viscous interactions, also has a substantial effect on the dynamics of the system (Fig. 7 ▸). At low viscosities, the initial decay in the intermediate scattering function is very fast and the amplitude of the oscillations is large. With increasing viscosity, the system becomes more damped such that the initial decay becomes much slower, the oscillation amplitude decreases and the height of the plateau increases. The small peak in the oscillation amplitude at η_3_ ≃ 0.001 Pa s is an artefact of the fitting procedure, as the oscillating part of the fit function has a uniform amplitude and does not capture decaying amplitude that is present in the simulations.

The strong effect of the layer oscillation amplitude is also useful from an experimental perspective. In contrast to the layer compression modulus, the layer sliding viscosity cannot be determined from X-ray or neutron reflectivity measurements. In a recent GINSES study of the effects of salt concentration on the behaviour of a lipid membrane stack, the *MembaneDyn* program was used to show that the layer sliding viscosity decreases with the addition of NaCl (Jaksch *et al.*, 2021 ▸).

### 3.5. Layer bending modulus: κ

**Figure 8:**
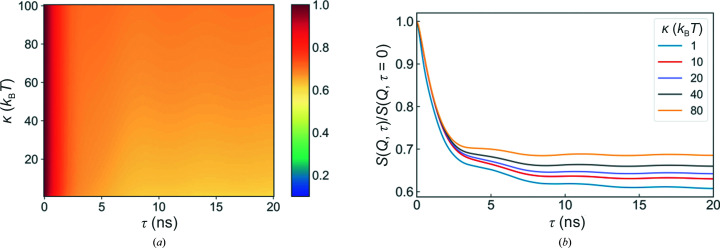
Overview of the simulation results for different layer bending moduli. (*a*) The evolution of the intermediate scattering functions with changing κ and (*b*) some selected examples. The standard parameter values used can be found in Table 1 ▸.

Fig. 8 ▸ shows how the dynamics are affected by the layer bending modulus κ, which is related to the bulk modulus *K* by *K* = κ/*d*_layer_. As already recognized in the original work by Romanov and Ul’yanov, the bending modulus has only a very minimal effect on the dynamics of the supported multilayer system. The height of the underlying plateau increases slightly with increasing κ; however, this effect is very small and can be neglected in the range of interest for most systems.

## 4. Discussion and conclusions

In this work, we have extended the mathematical framework to calculate the static scattering function from a supported membrane stack, first developed by Romanov and Ul’yanov, into the time domain, yielding the normalized intermediate scattering function *S*(*Q*, τ)/*S*(*Q*, τ = 0). This is a quantity that we can access experimentally via neutron spin-echo spectroscopy under grazing-incidence conditions. From the examples given above, the strongest oscillations are observed in systems with a small number of layers, a low layer compression modulus and a low layer viscosity. In addition, the oscillatory behaviour is best observed at small in-plane scattering vectors (*Q*_⊥_ < 0.008 Å), corresponding to large real-space length scales, and at out-of-plane scattering vectors *Q*_*z*_ on the shoulder of a Bragg peak.

**Figure 9:**
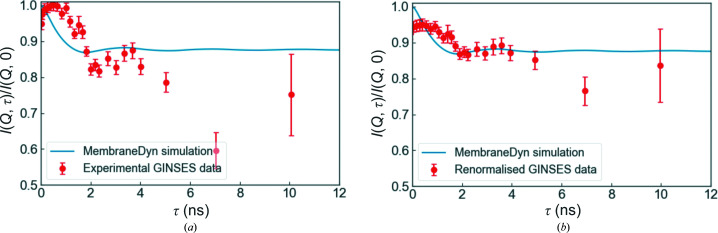
(*a*) Comparison of the experimental data (previously published in Jaksch *et al.*, 2017 ▸) with a *MembraneDyn* simulation. The parameters are given in Table 1 ▸, with the following exceptions: *N* = 10, *B* = 5 × 10^6^ N m^−2^, η_3_ = 1 × 10^−3^ Pa s. In (*b*) the experimental data have undergone a different background subtraction and normalization. Note that this is simply an illustration of how small differences in background subtraction and normalization can affect the data; it is not a fit.

In practice, previously published experimental GINSES data suggest that collective dynamics in supported membranes are more prominent than the *MembraneDyn* simulations predict (see Fig. 9 ▸ *a*). The reasons for this discrepancy may arise in part due to an oversimplification of the scattering function. The *MembraneDyn* program treats each layer as a thin membrane sheet, ignoring the thickness of the layers, the scattering length density (SLD) contrast and the form factor of the layers. This is not unreasonable as the ISF is normalized to unity and the measurement times are so long that only a single scattering vector **Q** can be probed in a typical experiment. It cannot be ruled out, however, that the inclusion of these parameters (*i.e.* the thickness, form factor and contrast) is necessary to perform quantitative analyses and fits, despite the associated increase in computation times.

A further possible explanation for the discrepancy stems from experimental considerations. In a GINSES experiment, the measured intermediate scattering function is likely to be affected by contributions from the background of the measurement. In particular, due to the grazing-incidence geometry, it is not always straightforward to determine and separate the different contributions from the elastic (non­decaying) portion of the scattering function or the contribution from incoherent scattering. This may give rise to large errors in normalization and/or background subtraction. This point is illustrated graphically in Fig. 9 ▸(*b*), where the data have been subjected to a slightly different normalization and background subtraction. Note that the simulated dynamics in Figs. 9 ▸(*a*) and 9 ▸(*b*) are identical; the data have not been fitted. Such issues may be solved by optimization of the experimental methods as well as through the use of virtual GISANS experiments (for polymers at interfaces, see, for example, Kyrey *et al.*, 2021 ▸) in which these contributions can be simulated.

In addition to implementation of the thickness, form-factor and contrast contributions, there is also room for optimization with regard to the computation time. It is numerically rather demanding to solve the required integrations in real space and reciprocal space accurately due to the oscillating Bessel function in equation (9). Although the Gaussian cutoff allows the real-space integration to be implemented in a stable and reliable manner with sparse integration points, the bottleneck currently resides in the reciprocal-space integration step. Due to the high-frequency oscillations, this currently requires a large number of integration points (∼2000). Adaptive routines could be used to optimize the integration steps, but unfortunately these are incompatible with the current structure of the program and would require a significant overhaul. Further details of the integration steps and parameters used can be found in the supporting information. We consider that the *MembraneDyn* program may be used in a fully quantitative manner to fit and interpret experimental GINSES data, in particular once some (or all) of the abovementioned improvements have been implemented.

## 5. Code availability

The Fortran code for the description of membrane fluctuations at interfaces can be found at https://jugit.fz-juelich.de/neutrons/membranedyn. The repository also contains a Jupyter notebook with the Fortran routine imported as a module.

## 6. Related literature

The following reference is cited in the supporting information for this article: Uhlenbeck & Ornstein (1930 ▸).

## Supplementary Material

Implementation, additional results and integration parameters. DOI: 10.1107/S2059798322008701/lp5060sup1.pdf

## Figures and Tables

**Table 1:** Summary of the parameters and values used for the simulations shown in Section 3

Parameter	Description	Value	Unit
*N*	Number of layers	5	
*B*	Layer compression modulus	1 × 10^6^	N m^−2^
κ	Layer bending modulus	19	*k*_B_ *T*
η_3_	Layer sliding viscosity	2 × 10^−3^	Pa s
*T*	Temperature	308.15	K
*d*_layer_	Interlayer distance	60	Å
*Q*_*z*_	*z* component of the scattering vector	0.21	Å^−1^
*Q*_⊥_	In-plane component of the scattering vector	0.0065	Å^−1^
*d*_ev_	Evanescent field depth	500	Å
*r*_max_	*r* integration limit	2000	Å
*q*_max_	*q* integration limit	1	Å^−1^
*N*_*r*_	Number of *r* integration points	2000	
*N*_*q*_	Number of *q* integration points	200	
*w*_cut_	Cutoff width	0.3	
