# Supplementary material for: MembraneDyn: simulating the dynamics of supported membrane stacks on the nanosecond timescale
Source: Acta Crystallogr D Struct Biol. 2022 Sep 27;78(Pt 10):1249–58. doi: 10.1107/S2059798322008701 (PMC9527762; doi:10.1107/S2059798322008701)
Supplement: Supplementary file 1 [file d-78-01249-sup1.pdf]

STRUCTURAL
BIOLOGY

Volume 78 (2022)

Supporting information for article:

MembraneDyn: simulating the dynamics of supported membrane stacks on the nanosecond timescale

Dominic W., Sebastian, Margarita, Purushottam, Henrich, Olaf, Michael, Dominic, Sebastian, Margarita, Purushottam, Henrich, Olaf and Michael

.

1. Implementation

In principle, the implementation of the Romanov model in the MembraneDyn software follows the theory outlined above. However, a number of changes have been made to complement and extend its functions. The most important of these, is the introduction of a time-dependence for the displacement amplitude correlation. This follows the form of the mean positional deviation of a damped harmonic oscillator from its original position, x_0, after time t (Uhlenbeck & Ornstein, 1930):

$$\overline{\overline{x}}^{x_0} = x_0 e^{-\frac{\beta}{2}t} \left(\frac{\beta}{2\omega_1} \sin \omega_1 t + \cos \omega_1 t \right) \tag{S1}$$

Where β is the damping coefficient and ω_1 the undamped angular frequency of the oscillator. The coefficients, β and ω are:

$$\beta^{(l)} = \mathrm{Im}\left(\omega_+^{(l)} + \omega_-^{(l)}\right) = -\frac{\eta_3 q_\perp^2}{\rho} \tag{S2}$$

$$\omega_1^{(l)} = \frac{\omega_+^{(l)} - \omega_-^{(l)}}{2} = -\frac{1}{2\rho d_{\mathrm{layer}}} \sqrt{-d_{\mathrm{layer}}^2 \eta_3^2 q_\perp^4 - 4B\rho\lambda^{(l)}} \tag{S3}$$

where $\lambda^{(l)}$ is the eigenvalue for the mode l, determined by solving the linear homogeneous equations discussed in the previous section. The inclusion of the time-dependence therefore requires the correlation functions to be calculated separately for each mode.

The other important changes relate to the calculation of the scattering function and can be summarised as follows:

1. The SLD contrast term is omitted.
2. In its place, an evanescent decay term has been included, which reflects the experimental conditions in GISANS and GINSES experiments.
3. A Gaussian cut-off term has been added to the layer displacement correlation function G_{nm}.

The contrast term, shown in Eq. 8a in the main text, is not included in the MembraneDyn program as it does not affect the normalised intermediate scattering function ($^{S(Q,\tau)}/_{S(Q,0)}$). The omission of this term therefore increases the computation speed. The evanescent (i.e. exponential) decay term is required for comparison with experimental data. In a GINSES experiment, the neutron beam undergoes total internal reflection at the interface between the silicon block and the sample, and the sample is only probed by the evanescent neutron field. In practice, the (neutron) refractive indices of the sample and the support differ only by a fraction of a percent and the penetration depth is fairly large (on the order of 100 nm). Nevertheless, the fact that the layers closer to the interface contribute more to the measured dynamics than those further away, must still be accounted for. This is done via the following evanescent term:

$$f_{\mathrm{ev}}(n,m) = \exp\left(-\frac{(N-n)\cdot d_{\mathrm{layer}}}{d_{\mathrm{ev}}}\right)\exp\left(-\frac{(N-m)\cdot d_{\mathrm{layer}}}{d_{\mathrm{ev}}}\right) \tag{S4}$$

where $d_{\rm ev}$ is the penetration depth. The effect of the evanescent term for $m = 0$ is shown in Figure S1. Note that whilst the displacement correlations for layers far away from the interface do not *explicitly* contribute to the scattering functions, the mere presence of layers above those probed, does affect the scattering implicitly by altering the dynamics of the system as a whole.

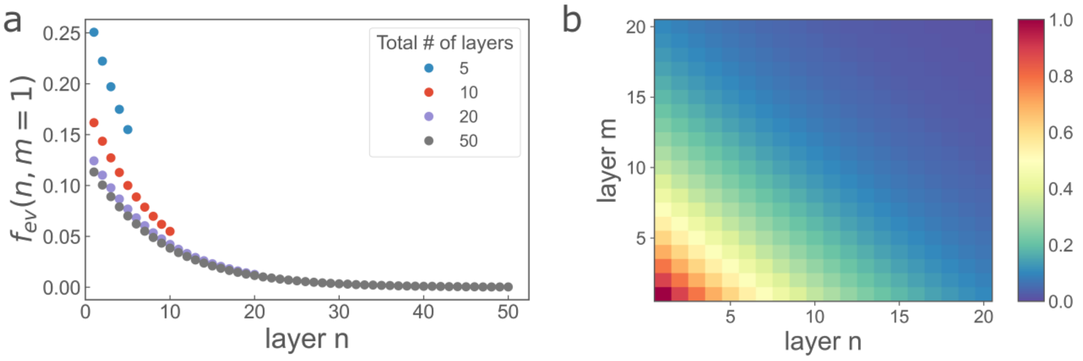

Fig. S1. Relative strengths of the contributions arising from the correlation functions u_{nm}, as dictated by the evanescent term in Eq. S4. (a) Shows the relative contributions from u_{n1} for different numbers of total layers, with the normalised total contribution for each system of N layers equal to unity. (b) Shows the relative contributions for a system with 20 layers normalised to the contribution from u_{11}. In both cases $d_{\rm ev} = 50$ nm.

Finally, the 'cut-off' term is required, as the scattering function is evaluated over a finite summation range, here Λ in Eq. 9 in the main text. Neglecting this term leads to artefacts such as spurious oscillations and negative scattering intensities and it is therefore necessary for comparison with experimental data. In this case, a Gaussian cut-off is used:

$$f_{\rm cut}(r) = \exp\left[-\left(\frac{r}{w_{\rm cut} r_{\rm max}}\right)^2\right] \tag{S5}$$

where $w_{\rm cut}$ is the normalised standard deviation of the Gaussian distribution (i.e. a value between zero and one). Other cut-off functions were also examined, such as the autocorrelation of a disc function:

$$f_{\rm cut}(r) = 1 - \frac{2r}{\pi r_{\rm max}}\sqrt{1 - \frac{r^2}{r_{\rm max}^2}} - \frac{2}{\pi}\sin^{-1}\left(\frac{r}{r_{\rm max}}\right) \tag{S6}$$

However, this was also found to yield some off-specular artefacts. The cut-off functions and their effects on the scattering intensity can be seen in Figure S2.

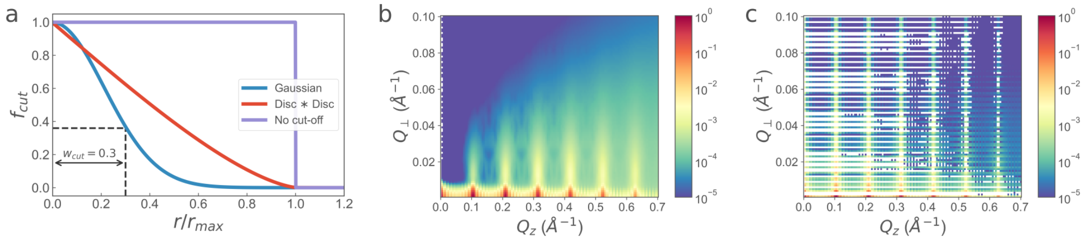

Fig. S2. (a) Possible cut-off functions for the numerical integration in real-space. (b) Shows the normalised scattering intensity at $\tau = 0$ with the Gaussian cut-off term applied. (c) Shows the normalised scattering intensity at $\tau = 0$ with no cut-off term applied. The white areas represent negative scattering intensity.

The calculation of the scattering function (Eq. 8 in the main text) is performed in three parts. As can be seen from Eqs. 7 and 9 in the main text, the function includes a multiple integral of the form:

$$\int_{r_{min}}^{r_{max}} \mathrm{d}r_\perp f(r_\perp) \exp\left(\int_{q_{min}}^{q_{max}} \mathrm{d}q_\perp g(q_\perp, r_\perp)\right) \tag{S7}$$

This can be solved numerically in two steps by first defining all integration points in $q_\perp$ and $r_\perp$ (note: both scalar values). The value of the inner integral is then calculated using the trapezoidal rule in a nested loop for each value of m, n, $r_\perp$ and t and stored in an array. The outer integral, over $r_\perp$, is solved in the second step, again using the trapezoidal rule, using the stored values from the previous step. Finally the summation steps are performed over n and m. The advantage of performing the multiple integral over two steps, is that the calculations do not require repeated function calls to external integration routines. As a result, the individual calculations can be performed relatively efficiently. The slight downside to this approach, is that the integration points must be defined in advance which precludes the use adaptive integration routines. As the functions become highly oscillatory at large $q_\perp$ and $r_\perp$ values,

extreme care must be taken when defining the limits and number of integration points (see Supporting Information for more details).

The equations above were first implemented as a stand-alone executable written in FORTRAN. A python extension module was then constructed using the FORTRAN source code in conjunction with the f2py command line tool. This module was then employed to perform the fits and simulations shown in the following section. Aside from the physical parameters of the model, which have been introduced above and are explored in more detail in below, the program also contains the option to modify a number of parameters governing the internal computation. The parameters $r_{\max}$ and N_r control the limits and number of points in the numerical integration over $r_\perp$, similarly, the parameters $q_{\max}$ and N_q govern the limits and number of points in the numerical integration over $q_\perp$. Finally, the parameter w_{cut} can be modified to adjust the width of the Gaussian used for the cut-off term (see Eq. S5).

With the exception of the cut-off term, all of the control parameters represent trade-offs between the accuracy of the simulation and the computation time. The integration interval must be wide enough and the integration points close enough together to capture all of the relevant behaviour. The integration parameters are examined in further detail below.

2. Additional Results

2.1. Layer Spacing: d_{layer}

Finally, Figure S3 demonstrates the effects of an evolving interlayer spacing. By comparison with Figure 3 in the main text, it can be seen that the changes in the observed dynamics are not directly linked to the interlaying spacing itself, but rather by the relationship between d and Q_z. When the system is no longer being probed on the shoulder of a correlation peak, the ISF decays much faster and the oscillations

associated with the collective motion become much weaker. Although these results are therefore a logical consequence of the results in section 3.1 of the main text, they are included to highlight the sensitivity of the system to very small changes in sample thickness (as can be seen in Figure S3b). This effect is thought to be a consequence of the fact that the layers are infinitely thin. In practice, this is obviously not the case and the dynamics are not expected to be this sensitive to the interlayer distance in real samples. It does demonstrate, however, that experimental sample should be well characterised and Q_z carefully chosen before any GINSES measurements.

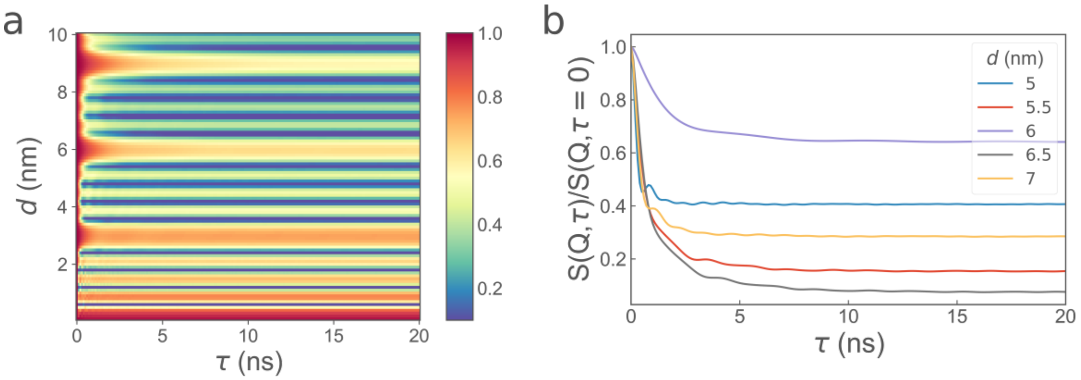

Fig. S3. (a) The evolution of the intermediate scattering functions with changing layer spacing and (b) examples demonstrating the sensitivity of the ISF to d_{layer}. The standard parameter values used can be found in Table 1 in the main text.

3. Integration Parameters

As noted in the main article, in order to obtain accurate results it is critical to select a suitable set of integration parameters. The two integration steps (over q and r respectively) take the form:

$$G = \int_0^{r_{\max}} \mathrm{d}r_\perp\, f(r_\perp) \exp\left(\int_0^{q_{\max}} \mathrm{d}q_\perp\, g(r_\perp, q_\perp)\right) \quad \text{(S8)}$$

To perform this calculation numerically, it is necessary to perform an integration over q for each integration point on r. In order to optimise the performance, the

integration over q is first carried out for all values of r and stored in an array. The subsequent integration over r then uses the stored values to calculate G. Whilst this approach reduces the number of function calls and allows the numerical integration steps to be performed quickly, it also requires that the integration points be determined in advance and therefore does not allow for the use of adaptive integration routines. This is significant, as the integrands can, in both cases, be highly oscillatory. In order to optimise the performance whilst maintaining accuracy, we have therefore investigated and characterised the response of both integrands with respect to the upper bounds and the number of integration points necessary to accurately capture the behaviour of the system. The data in the following plots were generated using the *Mathematica* software package.

3.1. Integration over q

At time $t = 0$, the q-integrand in Eq. S8 is given by:

$$g(r_\perp, q_\perp)_{nm} = q_\perp J_0(q_\perp r_\perp)(\hat{M}^{-1})_{nm}(q_\perp) \tag{S9}$$

where $\hat{M}$ is a matrix defined by u,v,x,y and z (as defined in the main text). Although the program calculates the above expression slightly differently (via summation of the individual modes) due to the time-dependence, the behaviour of the function with respect to $r_\perp$ and $q_\perp$, as shown in Figure S4, is identical. For small values of $r_\perp$ below $r_\perp \sim 10$ Å, the Bessel-term is approximately equal to 1 and Eq. S9 takes the form of a single, broad peak. As $r_\perp$ increases beyond ~ 10 Å, the integrand begins to oscillate with increasing frequency. For all combinations of n and m and all physically relevant parameter values (i.e. all of those investigated in the main article) the value of the integrand converges to zero at high $q_\perp$ (i.e. $q_{max} < 0.2$ Å^{-1}). Finally, in Figure SS4c, it can be seen that the number of q-integration points is highly dependent on $r_{\max}$. For large values of $r_{\max}$, at least 1000 points are required otherwise the calculated

scattering functions may be incorrect. For the calculated data shown in the main text, 2000 q-integration points were used with $q_{\max} = 0.2$ Å^{-1}.

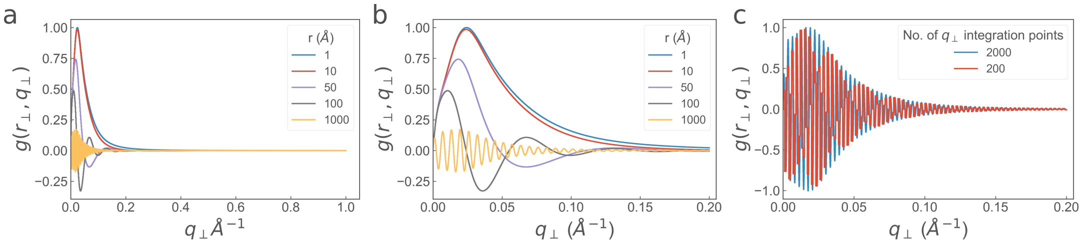

Fig. S4. (a) Calculation of the integrand of the inner integral in Eq. S8, corresponding to the RHS of Eq. S9 with $q_{\max} = 1$ Å^{-1}. (b) RHS of Eq. S9 with $q_{\max} = 0.2$ Å^{-1}. (c) RHS of Eq. S9 for $r = 2000$ Å with $q_{max} = 0.2$ Å^{-1}, highlighting the importance of including sufficient integration points.

3.2. Integration over r

Figure S5a shows the behaviour of the outer integral ($r_\perp$) in Eq. S8. The integral is clearly divergent (which causes the negative scattering intensities seen in Figure 3 in the main text) and this highlights need for a gradual 'cut-off' function. In this case, a Gaussian cut-off function is used (discussed in more detail in the main text) which adds a further parameter, r_{cut}, defining the strength or abruptness of the cut-off. When r_{cut} is large (~ 1), the integrand remains divergent, when r_{cut} is small (~ 0.1) the oscillatory nature of the function is lost. In the main text and in the following plots, a value of $r_{\text{cut}} = 0.3$ has been selected as this represents the largest possible value of r_{cut} that still exhibits convergent behaviour in the scattering function. As alluded to in the previous section, the value of $r_{\max}$ also has a significant influence on the behaviour of the integrand. Experimentally, $r_{\max}$ corresponds to the radius of the membrane patch being observed and consequently represents a maximum observable feature size. In the GINSES experiments performed to date, this size is approximately 100 nm, however, to account for the cut-off function, in this work we have opted to work with a value of $r_{\max} = 2000$ Å. In contrast to the $q_\perp$ integrand, the frequency of

the $r_\perp$ integrand is constant and depends only on $Q_\perp$ (as can be seen in Figure S5b). As this value is small (~ 0.0075 Å^{-1}), the number of integration points does not need to be as high as for the $q_\perp$ integral. For the calculated data shown in the main text, 200 r-integration points were used.

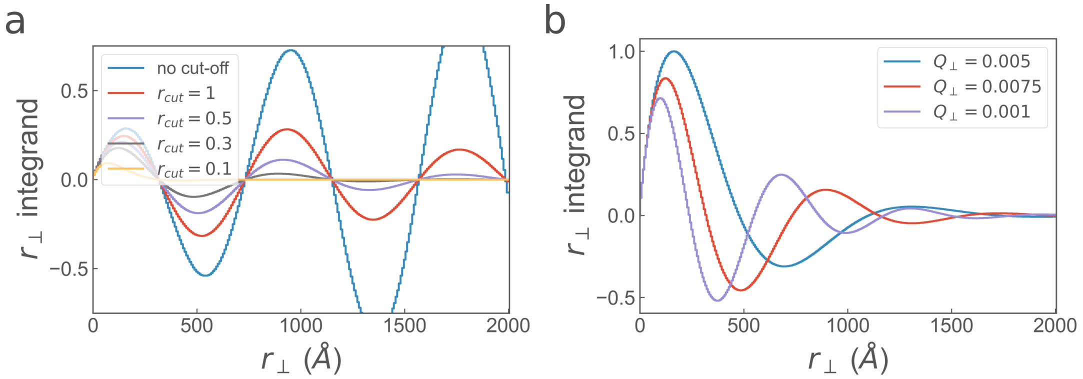

Fig. S5. (a) Calculation of the integrand of the outer integral in Eq. S8 for $q_{\max} = 0.2$ and $Q_\perp = 0.0075$ showing the effect of the $r_{\rm cut}$ parameter. (b) Calculation of the integrand of the outer integral in Eq. S8, showing the effect of the $Q_\perp$ parameter on the oscillation frequency.

References

Uhlenbeck, G. E. & Ornstein, L. S. (1930). *Physical Review*, **36**(5), 823–841.
URL: *https://link.aps.org/doi/10.1103/PhysRev.36.823*
